# Antibiotic Heteroresistance in *Klebsiella pneumoniae*

**DOI:** 10.3390/ijms23010449

**Published:** 2021-12-31

**Authors:** Karolina Stojowska-Swędrzyńska, Adrianna Łupkowska, Dorota Kuczyńska-Wiśnik, Ewa Laskowska

**Affiliations:** Department of General and Medical Biochemistry, Faculty of Biology, University of Gdansk, Wita Stwosza 59, 80-308 Gdansk, Poland; karolina.stojowska-swedrzynska@ug.edu.pl (K.S.-S.); adrianna.lupkowska@phdstud.ug.edu.pl (A.Ł.); dorota.kuczynska-wisnik@ug.edu.pl (D.K.-W.)

**Keywords:** *Klebsiella pneumoniae*, antibiotic heteroresistance, multidrug resistance

## Abstract

*Klebsiella pneumoniae* is one of the most common pathogens responsible for infections, including pneumonia, urinary tract infections, and bacteremias. The increasing prevalence of multidrug-resistant *K. pneumoniae* was recognized in 2017 by the World Health Organization as a critical public health threat. Heteroresistance, defined as the presence of a subpopulation of cells with a higher MIC than the dominant population, is a frequent phenotype in many pathogens. Numerous reports on heteroresistant *K. pneumoniae* isolates have been published in the last few years. Heteroresistance is difficult to detect and study due to its phenotypic and genetic instability. Recent findings provide strong evidence that heteroresistance may be associated with an increased risk of recurrent infections and antibiotic treatment failure. This review focuses on antibiotic heteroresistance mechanisms in *K. pneumoniae* and potential therapeutic strategies against antibiotic heteroresistant isolates.

## 1. Introduction

*Klebsiella pneumoniae* is a Gram-negative, encapsulated, non-motile bacterium commonly present in the environment and primarily associated with community-acquired pneumonia in patients with alcohol use disorder or diabetes mellitus [1,2]. In healthy humans, *K. pneumoniae* colonizes mucosal surfaces of the oropharynx, nasopharynx, and gastrointestinal tract [1,2]. Classical *K. pneumonia* (cKP), naturally resistant to ampicillin, carbenicillin, and ticarcillin due to the production of a chromosomal penicillinase (SHV-1), is becoming increasingly multidrug-resistant via the acquisition of resistance determinants carried on the chromosome or mobile elements (plasmids and transposons) [2,3,4]. Multidrug-resistant (MDR) cKP is one of the main factors responsible for nosocomial infections, including pneumonia, urinary tract, and bloodstream infections. MDR cKP producing extended-spectrum β-lactamases (ESBL) and carbapenemases are responsible for high mortality rates (40–50%), mainly among critically ill and immunocompromised patients [3]. The continuous accumulation of antibiotic-resistant genes in cKP has resulted in the emergence of extensively drug-resistant (XDR) and pandrug-resistant (PDR) strains with “super resistome”, which protects cKP against all available antimicrobials [5,6,7]. Over the last few years, an increasing number of hypervirulent *K. pneumoniae* (hvKP) isolates expressing a hypermucous phenotype have been repeatedly reported. The hvKP isolates possess a thick capsule and are more resistant to phagocytosis, complement-, and neutrophil-mediated activities than cKP [4]. It has been suggested that hvKP strains could survive within macrophages and neutrophils. Moreover, hvKp can use neutrophils as vehicles, in line with the “Trojan horse” infection mechanism, leading to disseminated infections [4]. Moreover, hvKP causes highly invasive infections in healthy and immunocompromised individuals, such as pyogenic liver abscess, endophthalmitis, and meningitis, [2,3,4,8,9,10,11,12]. The convergence of resistance and hypervirulence factors on one or multiple coexisting plasmids has resulted in the evolution of MDR and XDR hvKP isolates, which are now recognized as a severe threat to public health [4,13].

Due to life-threatening or deadly infections, *K. pneumoniae* was classified as the ESKAPE pathogen, an acronym for the *Enterococcus faecium*, *Staphylococcus aureus*, *K. pneumoniae*, *Acinetobacter baumannii*, *Pseudomonas aeruginosa*, and *Enterobacter* species. The ECAPE pathogens cause most nosocomial infections and are capable of “escaping” conventional antibiotic treatments [14].

Recent experimental and clinical studies suggest that the lack of efficient drugs against MDR, XDR, or PDR *K. pneumoniae* is not the only cause of treatment failures. Poor treatment outcomes may also result from the phenomenon of heteroresistance, which often leads to misdiagnoses and the inappropriate use of antibiotics.

## 2. Heteroresistance: Definition, Detection Methods, and Underlying Mechanisms

Antibiotic heteroresistance is a frequent phenotype observed in a wide range of pathogenic bacteria [15,16,17,18]. The terms “polyclonal” and “monoclonal” heteroresistance have been used to describe different forms of bacterial behavior in the presence of antibiotics [16]. In the case of polyclonal heteroresistance, genetically distinct clones exhibit stable resistant or susceptible phenotypes. Examples of polyclonal heteroresistance are co-infections by at least two isolates with different MIC (minimum inhibitory concentration) values or the emergence of stable resistant mutants during antibiotic treatment. In monoclonal heteroresistance, a subpopulation displays increased resistance transiently. Although this phenotype could be maintained for several generations, the resistant subpopulation often reverts to a susceptible phenotype. In the case of monoclonal heteroresistance, any resistant or susceptible cell may give rise to a new heteroresistant population [16,17]. The recently proposed definition of heteroresistance denotes the presence of a resistant subpopulation with an MIC at least eightfold higher than the highest concentration of the drug that does not affect the growth of the main population [16,18]. Other criteria that should be considered to classify clinical isolates as heteroresistant are the growth of the resistant subpopulation above the clinical breakpoint and its high frequency (above 1 × 10^−7^) [16].

**Figure 1:**
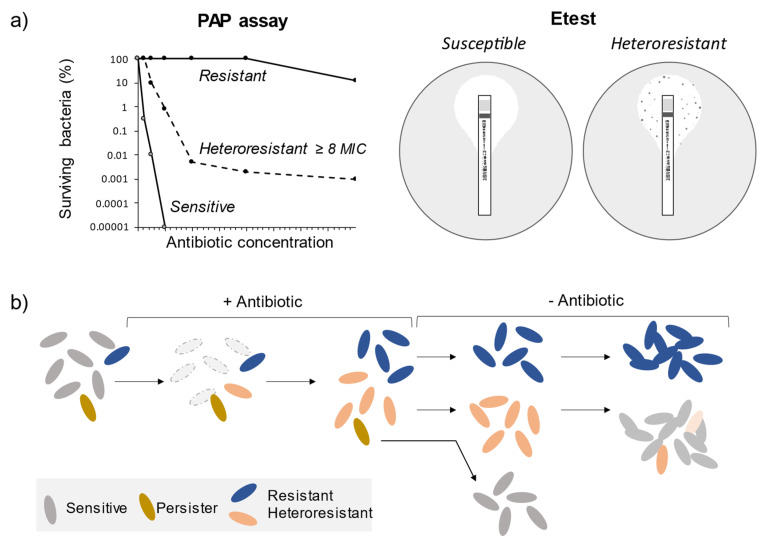
Heteroresistance detection methods and the difference between persister, resistant, and heteroresistant bacteria. (**a**) In the PAP assay, bacteria are spread on agar plates with increasing concentrations of antibiotics. Heteroresistance is determined as the growth of colonies at ≥8 × MIC of the main cell population. In the Etest assay, heteroresistance is determined by visible growth of colonies within the zone of clearing around the strips with a preformed continuous gradient of antibiotic concentrations. (**b**) Resistant bacteria that emerge before or during antibiotic treatment can grow in the presence of the antibiotic. They give rise to new populations distinct from the original ones. In the case of heteroresistance, resistant cells revert to the susceptible phenotype after the antibiotic treatment, usually due to high fitness costs. Persister cells can survive antibiotic treatment, but they cannot grow in the presence of the antibiotic. After the treatment, persisters can resume growth, switching back to the sensitive phenotype.

Due to phenotypic and genetic instability, heteroresistance is difficult to detect and study [16,18,19]. Rapid molecular and biochemical methods used to identify specific antibiotic resistance, such as real-time PCR or VITEK 2, are insufficient to detect heteroresistance due to the lack of a genetic marker or low sensitivity. The most reliable and quantitative method for detecting heteroresistant strains is the population analysis profile (PAP) assay. To assess the frequency of resistant mutants, bacteria are spread on agar plates with increasing concentrations of antibiotics. A gradual decrease in the number of CFU instead of a single-step loss of survival indicates the presence of a resistant subpopulation (Figure 1a). Unfortunately, due to the duration and complexity of the PAP assay, this method is not feasible for clinical use, and heteroresistance often remains undetected [15,20]. Alternative methods to PAP are the disc diffusion assay and Etests (Figure 1a). In the case of heteroresistant isolates, resistant colonies appear within the clearing zone around the disk or Etest strips. Both methods’ limitations are that they are not quantitative and may often give false-positive or false-negative results [21,22].

Various advanced techniques have been adapted to evaluate heteroresistant bacteria in recent years. The correct identification of heteroresistance can be made possible by comparing the growth rates of many single cells at several antibiotic concentrations. Testing only a single colony of the primary bacteria population isolated from patients may lead to a misinterpretation and poor assessment of the sensitivity of the population [23]. Therefore, direct single-cell imaging combined with microfluidics could be a promising method for the rapid detection of heteroresistant cells, as shown by Baltekin et al. [24]. Whole-genome sequencing is an increasingly used method to detect resistant subpopulations in clinical isolates [25,26]. Bauer et al. developed an extended Raman-based antibiotic susceptibility test. This assay was based on monitoring the metabolic activity spectroscopically measured by the D_2_O uptake in two model bacteria, *E. coli* and *Enterococcus faecalis* [27]. To assess clarithromycin susceptibility in *Helicobacter pylori*, droplet digital PCR was applied using probes detecting the most common mutations of the 23S rRNA gene that cause resistance to clarithromycin [28].

It should be noted that heteroresistance is distinct from another form of population heterogeneity persistence, in which a small subpopulation of dormant cells is transiently resistant to antibiotics [29,30]. However, in contrast to heteroresistant populations, persisters are not able to replicate in the presence of antibiotics (Figure 1b). Although persisters are metabolically inactive, they may resume growth after the therapy causing recurrent infections.

The most common mechanisms underlying heteroresistance in Gram-negative bacteria are spontaneous tandem gene amplification [22,31,32,33]. For example, amplification of the *prmD* gene was observed in colistin-heteroresistant *Salmonella typhimurium* isolates [13]. PrmD is a positive regulator that induces the synthesis of proteins involved in the modification of lipid A (see next paragraph). The addition of an arabinose derivative and phosphoethanolamine decreases the net negative charge of lipid A, fortifies the outer membrane (OM), and leads to colistin resistance. Other studies have demonstrated that the unstable tobramycin resistance of *Acinetobacter baumannii* emerged due to the extensive RecA-dependent amplification of the *aadB* gene encoding an aminoglycoside adenylyltransferase [32]. Amplification of the *lepB* gene encoding the target of arylomicin, signal peptidase LepB, was recently detected in unstable arylomicin-heteroresistant *E. coli* subpopulations [33]. The number of *lepB* copies in different subpopulations was remarkably variable, from 1 to 50 copies. The upregulation of resistant genes may also result from mutations in the promoter regions. An insertion sequence (ISAba1) was found in the promoter region of a β-lactamase gene (*bla*_ADC_) in imipenem-heteroresistant *A. baumannii* isolates [34]. Other mechanisms responsible for heteroresistance are the overproduction of efflux pumps and reduced expression of porins [35,36,37,38,39,40]. For example, heteroresistance to tigecycline in *Salmonella enterica* was associated with an increased level of the RamA transcriptional factor, resulting in the overexpression of the AcrAB-TolC and OqxAB efflux pumps [35]. Other studies have indicated that the downregulation of the OprD porin and overexpression of the efflux systems contributed to carbapenem and imipenem heteroresistance in *Pseudomonas aeruginosa* [38,41].

Mutations associated with heteroresistance often cause a high fitness cost [42]. Therefore, compensatory mutations are selected in the absence of antibiotics, and bacteria revert to a sensitive phenotype. These pseudorevertants have a genotype different from that of the susceptible ancestral isolate, even though they show a similar phenotype. Intrinsic instability of gene amplifications additionally contributes to the transient character of heteroresistance.

Heteroresistance may lead to antibiotic treatment failure due to the misclassification of heteroresistant isolates as susceptible strains. Consequently, inappropriate therapies fail to eradicate infections and facilitate the selection of resistant mutants [19,20,43,44]. It has been demonstrated that different antibiotics at levels below the MIC of the susceptible clinical isolates of *E. coli* and *Salmonella enterica* can cause the enrichment of resistant subpopulations with an increased copy number of resistance genes [19]. Band et al. reported that cumulative exposure of carbapenem-resistant *Enterobacterales* to β-lactams led to the development of heteroresistant isolates, which were gradually displaced by resistant cells [45]. Exposure to antibiotics and heteroresistance may also facilitate evasion of the host immune response. Napier et al. found that pretreatment of heteroresistant *E. cloacae* isolates with colistin resulted in cross-resistance to the host antimicrobial lysozyme [46]. In contrast, the colistin-sensitive *E. cloacae* isolates were not protected from lysozyme. Band et al. also revealed that the frequencies of resistant *E. cloacae* subpopulations in macrophage-depleted mice were decreased, indicating that the innate immune response may promote the appearance of resistant cells [47]. Indeed, it was found that specific innate host immune components (H_2_O_2_, lysozyme, the murine cationic antimicrobial peptide CRAMP, and its human orthologue LL37) increased the level of colistin-resistant subpopulations in vitro and in vivo.

## 3. Prevalence and Mechanisms of Heteroresistance in *K. pneumoniae*

Heteroresistance in *K. pneumoniae* has been described in several reports, as summarized in Table 1. Some of these studies have provided information about the molecular basis of emerging heteroresistant populations. It is worth noting that, apart from the core genome (~2000 genes), *K. pneumoniae* possesses a large set of accessory genes located in the chromosome or plasmids (~3000–4000 genes) and encoding specific virulence factors and antibiotic resistance mechanisms. Its high variability may contribute to population heterogeneity and favor the formation of heteroresistant subpopulations [3,46].

### 3.1. Heteroresistance to Colistin

The most commonly reported examples of *K. pneumoniae* heteroresistance with known mechanisms are clinical isolates that produce polymixin-resistant subpopulations (Table 1). Two closely related polymyxin antibiotics, colistin (polymyxin E) and polymyxin B., are used clinically. Colistin is a drug of last resort for infections caused by *K. pneumoniae*. In general, colistin kills Gram-negative pathogens, targeting the lipid A moiety of lipopolysaccharide (LPS) and destabilizing the OM by displacing divalent cations Ca^2+^ and Mg^2+^ that form bridges between LPS [66]. According to a recently proposed model, colistin also targets LPS molecules during its transport from the cytoplasm to the inner membrane (IM) and then to the OM. This colistin activity results in IM permeabilization, loss of cytoplasm content, cell lysis, and death [67]. Colistin resistance can appear via different mechanisms, most of which result in the addition of cationic moieties, 4-amino-4-deoxy-L-arabinose (L-Ara4N), and phosphoethanolamine (PEtN) to lipid A. Consequently, positively charged polymyxins are repelled from the membrane [66,67,68]. Lipid A hydroxylation and palmitoylation have recently been proposed as an alternative mechanism of colistin resistance [68]. The anionic capsular polysaccharide significantly limits the access of polymyxins to the OM. Hence, *K. pneumoniae* acapsular mutants show increased susceptibility to polymyxins [66].

**Figure 2:**
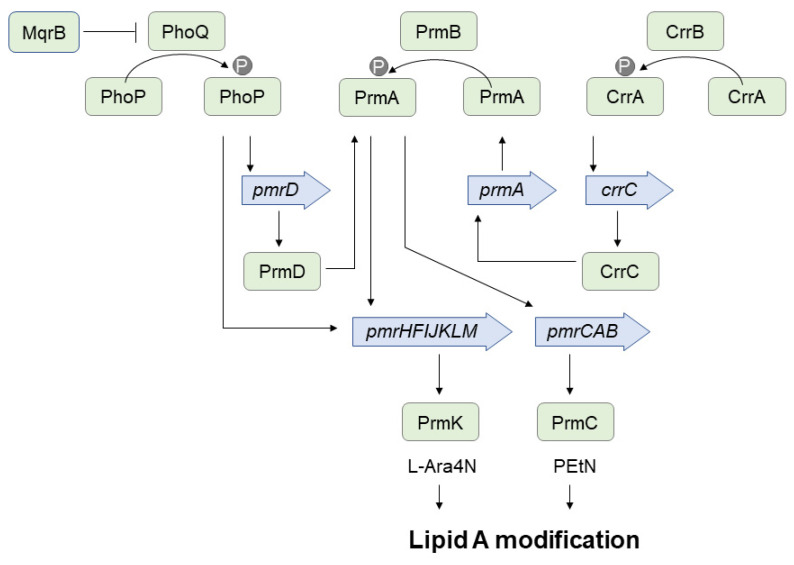
Two-component systems PhoPQ, PmrAB, and CrrAB control lipid A modifications by adding 4-amino-4-deoxy-l-arabinose (L-Ara4N) and phosphoethanolamine (PEtN). The modifications decrease the net negative charge of the lipid A, resulting in colistin resistance. See the text for more details.

In the first study that revealed mechanisms responsible for colistin heteroresistance in *K. pneumoniae*, Jayol et al. found that the colistin-resistant subpopulation harbored a single amino acid change (Asp191Tyr) in protein PhoP [50]. PhoP belongs to the PhoPQ two-component system, which is highly conserved among many Gram-negative bacteria (Figure 2). The sensor kinase PhoQ can respond by autophosphorylation to several environmental changes, including low pH, low Mg^2+^ concentrations, and the presence of antimicrobial peptides. The phosphate group is then transferred to the response regulator PhoP, which activates the expression of downstream genes involved in LPS modifications (Figure 2). The observed amino acid substitution in PhoP in colistin-resistant *K. pneumoniae* subpopulations caused the overexpression of the *pmrHFIJKLM* operon (also known as *arnBCADTEF* or *pbgPE*) responsible for L-Ara4N synthesis [50]. The PrmK transferase catalases the last step of the modification. The reversion of colistin resistance to susceptible phenotype was associated with the inactivation of PhoP caused by a partial deletion in the *phoP* gene.

Several studies have confirmed the contribution of the PhoPQ system in colistin heteroresistance [44,51,56]. It was proposed that an amino acid change in the sensor domain of PhoQ or PhoQ overexpression may lead to the constitutive phosphorylation of PhoP, and the subsequent activation of genes involved in lipid A modifications [51]. Colistin heteroresistance in *K. pneumoniae* was also attributed to mutations in the *pmrAB* genes, encoding another two-component system that controls the synthesis of L-Ara4N and PEtN (Figure 2) [21,56,57]. It is worth noting that diverse resistant subpopulations with different amino acid substitutions in PhoPQ and PmrAB were detected in various isolates. Evolution experiments have shown that unstable colistin-heteroresistance in *K. pneumoniae* might also emerge due to mutations in the *crrB* gene [55]. The CrrAB two-component system is involved in the control of the *pmrHFIJKLM* operon via the PmrAB regulatory network (Figure 2). Other studies have found that clinical *crrB* mutations lead to the addition of both L-Ara4N and pEtN to lipid A, inducing higher polymyxin resistance [69]. Colistin resistance may also be linked to mutations, insertions, or deletions in the *mgrB*, *yciM*, and *lpxM* genes [44,51,53,57]. The *mgrB* gene encodes a small transmembrane protein which acts as a negative regulator of the PhoPQ system. Therefore, its inactivation leads to overexpression of the *pmrHFIJKLM* operon [44,53,57]. The inactivation of the *mgrB* gene may also result in a thicker multilayered capsule which protects bacteria against colistin [70]. It is worth noting that inactivation of *mgrB* by point mutations, insertions, or deletions is one of the most common colistin resistance mechanisms in *K. pneumoniae* isolated from patients treated with colistin [63,71]. The role of *yciM* and *lpxM* genes in the emergence of colistin-resistant *K. pneumoniae* subpopulations is probably associated with the regulation of LPS biosynthesis and lipid acetylation [51].

Colistin heteroresistance may also be linked to the emergence of a small colony variant phenotype during *K. pneumoniae* biofilm formation [52]. It has been demonstrated that these slow-growing colistin-resistant bacteria were absent in a planktonic population that was colistin-susceptible. Biofilms are multicellular communities embedded in extracellular polymeric substances that may constitute a physical barrier for antibiotics and other stress factors [72,73,74]. The complex structure of the biofilm facilitates the formation of heterogeneous subpopulations with different gene expression patterns. The innermost layers of the biofilm have limited access to oxygen and nutrients, which, in turn, may inhibit metabolism or induce specific pathways that enable bacteria to survive under stressful conditions, including antibiotics.

### 3.2. Heteroresistance to Carbapenems

The expression of ESBLs, AmpC-β-lactamases (DHA, FOX), and carbapenemases is the main cause of β-lactams resistance in *K. pneumoniae* [3,75]. Plasmid-based ESBLs belong to the CTX-M, SHV, and TEM families and hydrolyze third-generation cephalosporins and aztreonam. The main *K. pneumoniae* carbapenemase (KPC) is the most common class A serine β-lactamase, encoded by the *bla*_KPC_ gene carried on plasmids and transported in the Tn4401 transposon, which facilitates its efficient transfer between bacterial isolates [75]. The increasing number of new KPC variants encoded on mobile genetic elements and their dissemination in Gramm-negative bacteria has been recently reported [76,77]. In addition to KPC, *K. pneumoniae* may produce different metallo-β-lactamases, including New Delhi metallo-β-lactamase (NDM), Verona integron-encoded metallo-b-lactamase (VIM), imipenemase (IMP), and OXY-48-like carbapenemases [3,75]. The emergence of NDMs-producing *K. pneumoniae* is of great concern because such isolates exhibit multidrug resistance and spread rapidly. Multiple studies have demonstrated that the enhanced resistance of *K. pneumoniae* to β-lactams may also result from deletions or mutations within the major outer membrane porins OmpK35 and OmpK36. It was found that OmpK35 and OmpK36 produce larger and more permeable channels than their *E. coli* homologs OmpF and OmpC. When third-generation cephalosporins were introduced into clinical use, this difference explains why *K. pneumoniae* isolates were initially more sensitive to these antibiotics than *E. coli* strains [78]. Porin deficiency reduces the uptake of β-lactams and, in combination with ESBLs activity, may confer clinical resistance [40,62,79]. The emergence of imipenem-resistant subpopulations with decreased levels of OmpK36 was reported by Adams-Sapper et al. (Table 1). The imipenem MIC for the subpopulations increased at least 32-fold, providing that a carbapenemase encoded by the *bla*_KPC-2_ gene was expressed [58]. Individual isolates that lost OmpK36 upon imipenem exposure regained expression of the porin in drug-free media and reverted to the heteroresistant phenotype. However, most isolates lost OmpK36 permanently and maintained the high-level imipenem resistance. Pournaras et al. (2010) found that meropenem heteroresistance may result from the transient overexpression of the *bla*_KPC-2_ gene [61]. After daily passages on a meropenem-free medium, the resistant subpopulations reverted into meropenem susceptible strains.

### 3.3. Heteroresistance to Tetracyclines

Drug efflux and ribosomal protection are predominant mechanisms of tetracycline resistance in many Gram-negative bacteria [80]. To overcome this problem, new tetracycline derivatives—semisynthetic tigecycline and fully synthetic eravacycline—were developed [81]. In recent years, increasing numbers of tigecycline- and eravacycline-resistant *K. pneumoniae* isolates have been reported [82]. The resistant mechanisms include overproduction of the efflux pumps AcrAB, OqxAB, and MacAB. Expression of the *acrAB* and *oqxAB* genes is regulated by global transcriptional activators such as RamA, SoxS, and MarA, which are controlled by their transcriptional repressors RamR, SoxR, and MarR, respectively [39,82]. Tigecycline- and eravacycline-resistance is also caused by mutations in the ribosomal protein S10 (encoded by the *rpsJ* gene), which prevent the binding of the antibiotics to the small 30S ribosomal subunit [81].

So far, tigecycline- and eravacycline-heteroresistant isolates have been reported by three independent studies (Table 1) [39,63,64]. Multiple mechanisms have contributed to tigecycline resistance in heteroresistant subpopulations, including (1) the overexpression of the *acrAB* efflux pump genes; (2) mutations of the *ramR* gene, which resulted in the RamA upregulation and led to AcrAB-TolC efflux pump production; (3) mutation of the *soxR* gene, which resulted in the SoxS overexpression and AcrAB-TolC upregulation; (4) the nucleotide insertion and frameshifts in the *rpsJ* gene, which encodes the ribosomal S10 protein. These mutations might weaken tigecycline binding to 16S rRNA [63]. Other findings indicated that eravacycline heteroresistance might result from the overexpression of the OqxAB and MacAB efflux pumps and the transcriptional regulator RamA [39]. The heteroresistant eravacycline strains originated from the samples not pre-treated with tigecycline or erevacyclin. Therefore, the overexpression of AcrAB and OqzAB may indicate that the isolates were exposed to other compounds transported by these pumps.

### 3.4. Heteroresistance to Aminoglycosides

Aminoglycoside-modifying enzymes (AMEs) and the presence of 16S ribosomal RNA methyltransferases (*armA*, *rmtA*, *rmtB*, *rmtC*, *rmtD*, *rmtE*, and *npmA*) are the main cause of aminoglycoside resistance in *K. pneumoniae* and other *Enterobacterales* [83,84]. AMEs include N-acetyltransferases, O-nucleotidyltransferases, and O-phosphotransferases, which covalently modify specific amino or hydroxyl moieties on the aminoglycosides.

Aminoglycoside heteroresistance in *K. pneumoniae* isolates was mainly attributed to the amplification of the AMEs genes that were harbored on plasmids and encoded aminoglycosides acetyl-transferases (*aac*) (Table 1) [22]. In the presence of gentamycin, tobramycin, or netilmicin, the number of plasmid copies and *aac* copies per plasmid increased. The resistance phenotype was unstable, and after 40 generations without the pressure of antibiotics, the number of *aac* copies and plasmid copies decreased, or the plasmid was lost. Consequently, the MIC values reverted to that of the parental isolates. Amikacin-resistant subpopulations had mutations in the *ubiJ* and *cydA* genes, which are associated with a small colony variant phenotype or aminoglycoside resistance. During growth in the absence of amikacin, compensatory point mutations or duplications (*ubiJ*) were selected, leading to amikacin susceptibility of the revertants. The reversion to aminoglycoside susceptibility in the absence of selective pressure resulted from the fitness cost conferred by the *acc* gene amplification and amikacin resistance mutations [22]. It has also been found that amikacin heteroresistance can be associated with the unstable overexpression of *aac* genes and several mutations (SNVs, single nucleotide variations) in genes encoding proteins involved in various physiological processes, including carbohydrate uptake and chromosome segregation [60].

## 4. Therapeutic Strategies against Heteroresistant *K. pneumoniae*

Recent findings suggest that combinations of clinically approved antibiotics might eradicate infections associated with heteroresistant *K. pneumoniae* (Table 2) [56,64,85,86]. Cheong et al. found that meropenem combined with colistin might be a therapeutic option for infections caused by colistin-heteroresistant *K. pneumoniae* [56]. Meropenem alone eradicated a heteroresistant isolate, but its combination with colistin was more effective. Tian et al. investigated the combined effect of polymyxin B and tigecycline against *K. pneumoniae* isolates manifesting dual heteroresistance to both antibiotics [64]. As expected, antibiotic monotherapy (polymyxin B or tigecycline) had a transient inhibitory effect. Susceptible bacteria were killed, but resistant subpopulations were not eliminated. The combination of polymyxin B and tigecycline, used at reduced doses, successfully eradicated susceptible and resistant subpopulations. In other studies, the combination of ceftazidime/avibactam (β-lactam/β-lactamase inhibitor combination) improved the efficacy of polymyxin B against heteroresistant carbapenemase-producing *K. pneumoniae* isolates and hindered the emergence of polymyxin-resistant subpopulations in vitro [86]. Band et al. investigated different combinations of antibiotics against a *K. pneumoniae* isolate that was heteroresistant to colistin and fosfomycin [85]. Colistin and fosfomycin monotherapy did not inhibit infection in mice, but the combination of both drugs significantly reduced the level of bacteria. Another tested isolate exhibited homogenous resistance to colistin and heteroresistance to fosfomycin and ceftazidime. Only dual therapy with fosfomycin and ceftazidime eradicated the infection, while any combination with colistin (colistin + fosfomycin or colistin + ceftazidime) was not effective. These results indicate that multidrug therapies should use antibiotics targeting different heteroresistant subpopulations rather than antibiotics targeting homogeneously resistant populations. Further studies revealed that this approach resulted in decreased survival, even in the case of two pandrug-resistant *K. pneumoniae* isolates known to cause untreatable infections (Table 2) [85].

## 5. Concluding Remarks

Numerous recent studies have revealed that heteroresistance occurs relatively often in *K. pneumoniae* clinical isolates (Table 1). Mutations and mechanisms responsible for the emergence of heteroresistant *K. pneumoniae* subpopulations have been previously identified as antibiotic-resistant factors in *Klebsiella* and other *Enterobacteriaceae*. These mechanisms have often contributed to unstable heteroresistance and have included lipid A modifications, the overexpression of pump systems, or gene amplification.

Although it has been well documented that bacterial biofilms are highly heterogeneous populations that are more drug-resistant than planktonic bacteria, little is known about the influence of biofilm’s environment on heteroresistance. The presence of colistin-heteroresistant subpopulations in *K. pneumoniae* biofilms is the only example described so far [52,73]. It was found that planktonic cells were susceptible to colistin, whereas colistin resistance exclusively detected within the biofilm population was linked with the small colony variant phenotype. Contrary to these results, Zhang et al. demonstrated that slow growth and biofilm formation do not contribute to amikacin heteroresistance in *K. pneumoniae* [60].

It should be kept in mind that most experiments focused on heteroresistance have been performed in vitro or using animal models. Therefore, these results may not reflect the mechanisms occurring in the host organism. Many additional issues should be considered, e.g., the interplay between the pathogen and the host immune response, as well as the co-existence of several heterogeneous subpopulations, including persister and VBNC (*viable but non-culturable*) bacteria, which are difficult to detect [17]. The impact of heteroresistance on antibiotic treatment failure has been a matter of extensive debate. However, increasing data strongly suggest that heteroresistance may be responsible for poor outcomes and recurrent infections with pathogens classified as susceptible. The mathematical modeling of pharmacodynamics of heteroresistance in *K. pneumoniae* and other species supports this assumption [22]. Recurrent heteroresistant infections treated repeatedly with the same antibiotic may allow the evolution of drug-resistant bacteria [60]. Therefore, it is crucial to improve methods for detecting heteroresistance and develop effective therapeutic strategies, which should be based on a combination of antibiotics as suggested by recent studies.

It has been recently demonstrated that human contact restrictions and other strategies to prevent SARS-CoV-2 transmission during the COVID-19 pandemic have limited the spread of *K. pneumoniae* and other ESKAPE pathogens [87,88]. However, the extensive use of disinfectants and increased administration of antibiotics to prevent bacterial co-infections during the COVID-19 pandemic may enhance the emergence and spread of drug resistance [89,90,91]. Several studies have indicated that the prevalence of carbapenem-resistant *K. pneumoniae* co-infection in COVID-19 patients may reach more than 50% of cases [92,93]. Moreover, *K. pneumoniae* heteroresistance toward chlorhexidine, the most widely used disinfectant in hospitals, has been previously described (Table 1) [65]. Given the lack of standard procedures and the difficulties in detecting heteroresistance, further studies are urgently needed to understand the mechanisms underlying *K. pneumoniae* hetero resistance and prevent the spread of multidrug-resistant infections.

## Figures and Tables

**Table 1:** Examples of heteroresistance in *K. pneumoniae*.

Antibiotic		Sample	Prevalence of Heteroresistance	Mechanisms of Heteroresistance	Stability of Resistant Phenotypes	References
Colistin		Multidrug-resistant clinical isolates (worldwide)	15/21	ND	ND	[48]
		Carbapenemase-producing clinical strains (Greece)	12/20	ND	Stable (11) Unstable (1)	[49]
		Multidrug-resistant clinical isolate (South Africa)	1/1	Single amino acid change in PhoP, activation of the *pmrHFIJKLM* operon	Unstable	[50]
		ESBL-producing clinical isolates	5/13	Mutations in the *lpxM*, *mgrB*, *phoQ* and *yciM* genes	ND	[51]
		Urine clinical isolate (Portugal)	1/1	Biofilm, small colony variant phenotype	ND	[52]
		Stool clinical isolate (France)	1/1	Single nucleotide insertion in the *mreB* gene	Unstable	[53]
		Clinical isolates (Hungary)	68/140	ND	ND	[54]
		Colistin susceptible *K. pneu-moniae* Ecl8	1/1	Mutations in the *crrB* gene	Unstable	[55]
		Multidrug-resistant urine isolates (USA)	2/2	Lower expression of *mgrB*, higher expression of *phoP*	Stable	[44]
		Clinical blood isolates (South Korea)	3/252	Different amino acid substitutions in PmrAB and PhoPQ	ND	[56]
		ESBL-producing clinical isolates (Chile)	8/60	Diverse mutations in PmrAB and PhoPQ, disruption of the *mgrB* gene	Stable	[57]
		Clinical carbapenem-resistant isolates (South Korea)	12/12	Different amino acid substitutions in PmrAB and PhoPQ	Stable	[21]
Carbapenems	Imipenem	Clinical isolates (Brazil, USA)	8/15	Carriage of the *bla*_KPC_ gene, decreased expression of OmpK36	Stable (6) Unstable (2)	[58]
		VIM-1-producing isolates (Spain)	3/18	ND	ND	[59]
		Clinical isolates (China)	75/155	ND	ND	[60]
	Meropenem	Clinical isolates (Greece)	6/6	Overexpression of the *bla*_KPC-2_ gene	ND	[61]
		OXA-48-producing isolates (Spain)	24/24	Decreased OmpK36 expression or activity	Stable	[62]
		Clinical isolates (China)	38/155	ND	ND	[60]
Tetracyclines	Tigecycline	Clinical isolates (China)	21/334	Overexpression of the *acrAB* genes, mutations in the *ramR*, *soxR* and *rpsJ* genes	Stable	[63]
		Carbapenem-resistant clinical isolates (China)	49/95	Upregulated expression of the *pmrA*, *phoP* and *acrB* genes	ND	[64]
	Eravacycline	Clinical isolates (China)	20/393	Mutation in the *acrR* or *ramR* genes, overexpression of the MacAB efflux pump	ND	[39]
Aminoglycosides	Amikacin	Clinical isolates (China)	13/155	Increased expression of *aac*(6′)-*Ib*, *aph* (3′)-*Ia*, *aac* (3)-*II* Mutations in *parB*, threonine dehydrogenase, Ssb, Sid	Unstable (11) Stable (2)	[60]
	Amikacin	Clinical isolates (Sweden)	2/10	Mutations: *ubiJ* Δ10 nt, PTS glucose transporter subunit IIA Δ1 nt, *cydA*Δ57 nt	Unstable	[22]
	Gentamycin		2/10	*aac*(3)-IIa amplification		
	Tobramycin		3/10	*aac*(6′)-Ib-ct or *aac*(3)-IIa amplification		
	Netilmicin		4/10	*aac*(6′)-Ib-ct amplification		
Chlorhexidine		XDR clinical isolates (Israel)	113/126	ND	ND	[65]

Prevalence of heteroresistance, number of heteroresistant isolates/total number of isolates; ND, not determined.

**Table 2:** Drug combinations effective against heteroresistant *K. pneumonia* isolates.

Isolates	Drug Combination	References
MDR, colistin-heteroresistant	Colistin + meropenem	[56]
Carbapenem-resistant, heteroresistant to polymyxin B, and resistant, heteroresistant or susceptible to tigecycline (4 isolates)	Polymyxin B + tigecycline	[64]
Carbapenem-resistant, heteroresistant to colistin and fosfomycin	Colistin + fosfomycin	[85]
Carbapenem-resistant, heteroresistant to fosfomycin and ceftazidime	Fosfomycin + ceftazidime	
Pandrug-resistant, heteroresistant to fosfomycin and sulfamethoxazole/trimethoprim	Fosfomycin + sulfamethoxazole/trimethoprim	
Pandrug-resistant, heteroresistant to amikacin and piperacillin/tazobactam	Amikacin + piperacillin/tazobactam	
Carbapenem-resistant, heteroresistant to polymyxin B (4 isolates)	Polymyxin B + ceftazidime/avibactam	[86]

## Abbreviations

AMEs	Aminoglycoside modifying enzymes
cKP	Classical *Klebsiella pneumoniae*
ESBL	Extended-spectrum beta-lactamases
hvKP	Hypervirulent *Klebsiella pneumoniae*
IM	Inner membrane
L-Ara4N	4-amino-4-deoxy-L-arabinose
MDR	Multidrug-resistant
MIC	Minimum inhibitory concentration
OM	Outer membrane
PAP	Population analysis profile
PEtN	Phosphoethanolamine
PDR	Pandrug-resistant
SNVs	Single nucleotide variations
XDR	Extensively drug-resistant
